# Supplementary material for: Synapse-specific diversity of distinct postsynaptic GluN2 subtypes defines transmission strength in spinal lamina I
Source: Front Synaptic Neurosci. 2023 Jul 12;15:1197174. doi: 10.3389/fnsyn.2023.1197174 (PMC10368998; doi:10.3389/fnsyn.2023.1197174)

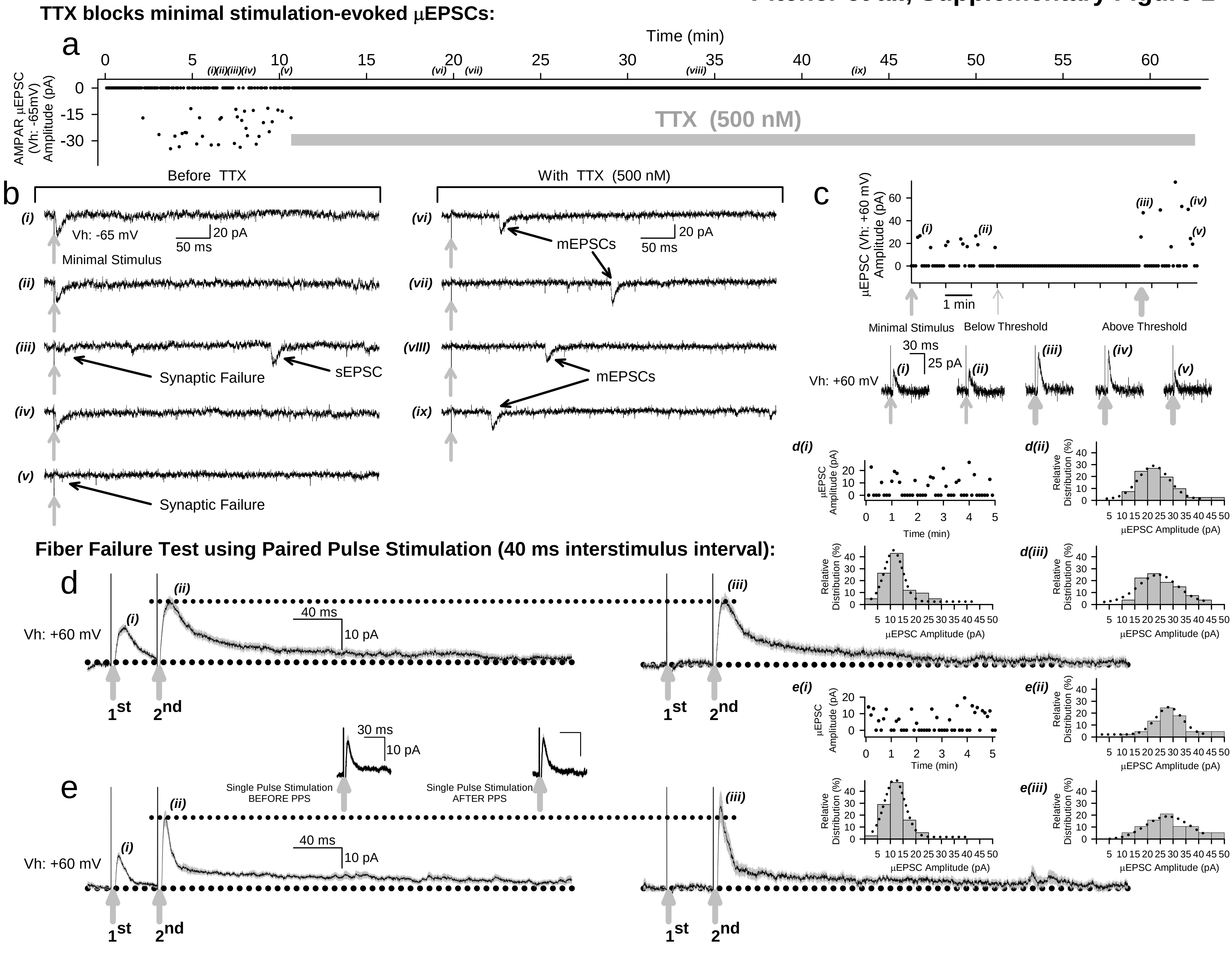

TTX blocks minimal stimulation-evoked μEPSCs:
a
Time (min)
AMPAR μEPSC (Vh: -65mV) Amplitude (pA)
TTX (500 nM)
b
Before TTX
With TTX (500 nM)
Vh: -65 mV
20 pA
50 ms
Minimal Stimulus
mEPSCs
Synaptic Failure
sEPSC
c
μEPSC (Vh: +60 mV) Amplitude (pA)
1 min
Minimal Stimulus
Below Threshold
Above Threshold
30 ms
25 pA
Vh: +60 mV
d(i)
d(ii)
d(iii)
μEPSC Amplitude (pA)
Time (min)
Relative Distribution (%)
Fiber Failure Test using Paired Pulse Stimulation (40 ms interstimulus interval):
d
e
Vh: +60 mV
40 ms
10 pA
1st
2nd
30 ms
Single Pulse Stimulation BEFORE PPS
Single Pulse Stimulation AFTER PPS
e(i)
e(ii)
e(iii)

Pitcher et al., . Supplementary Figure 2

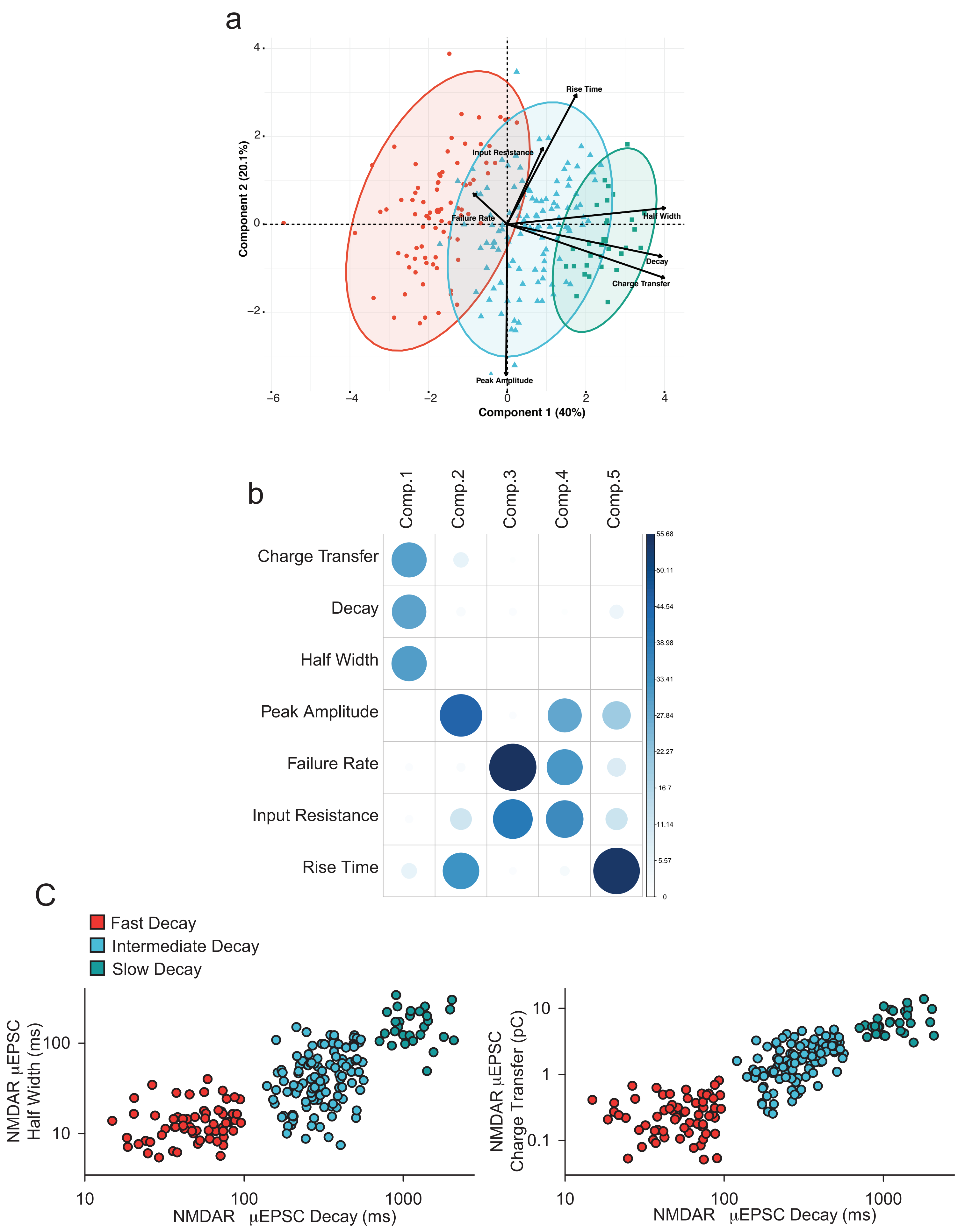

Pitcher et al., Supplementary Figure 3

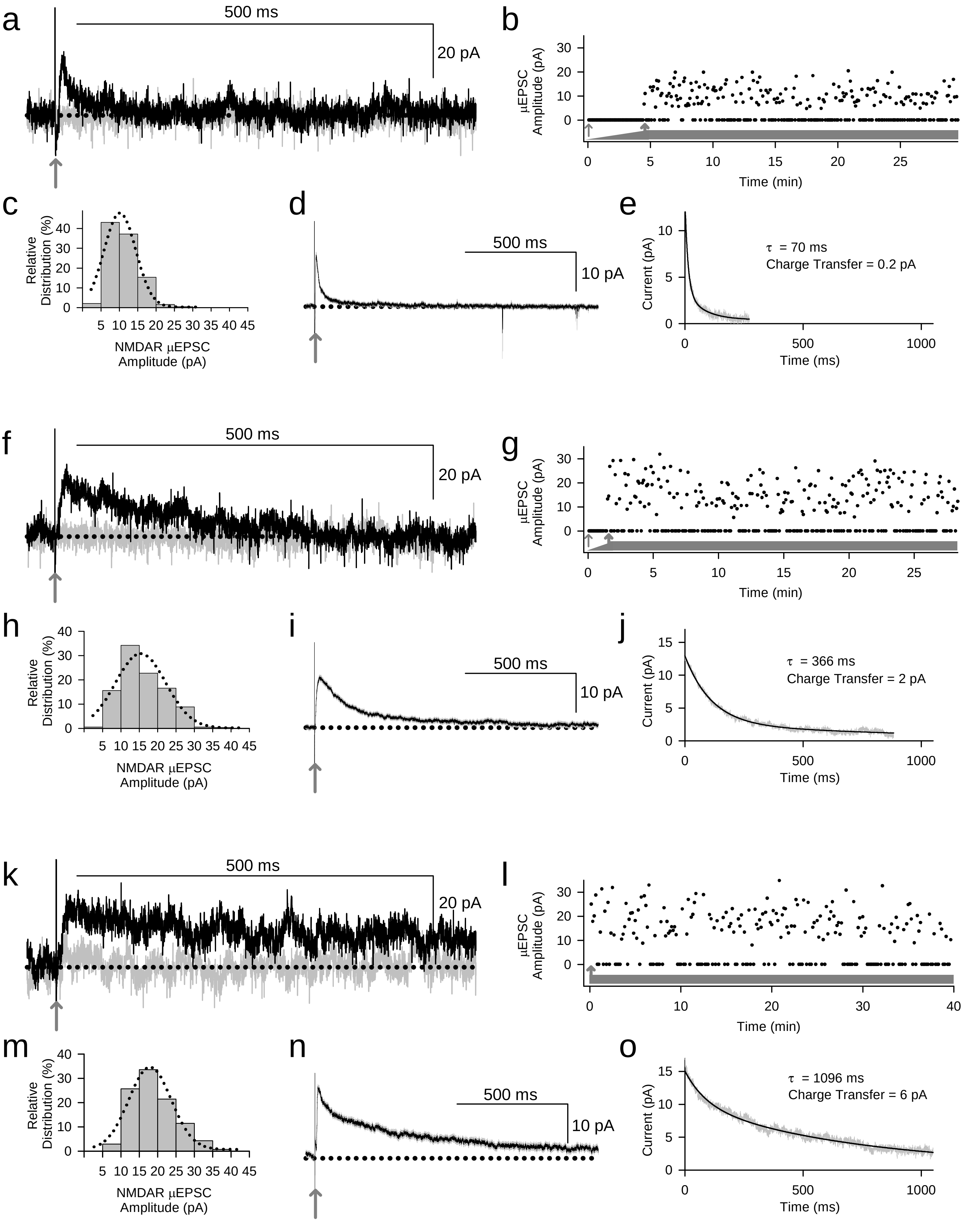

Pitcher et al., Supplementary Figure 4

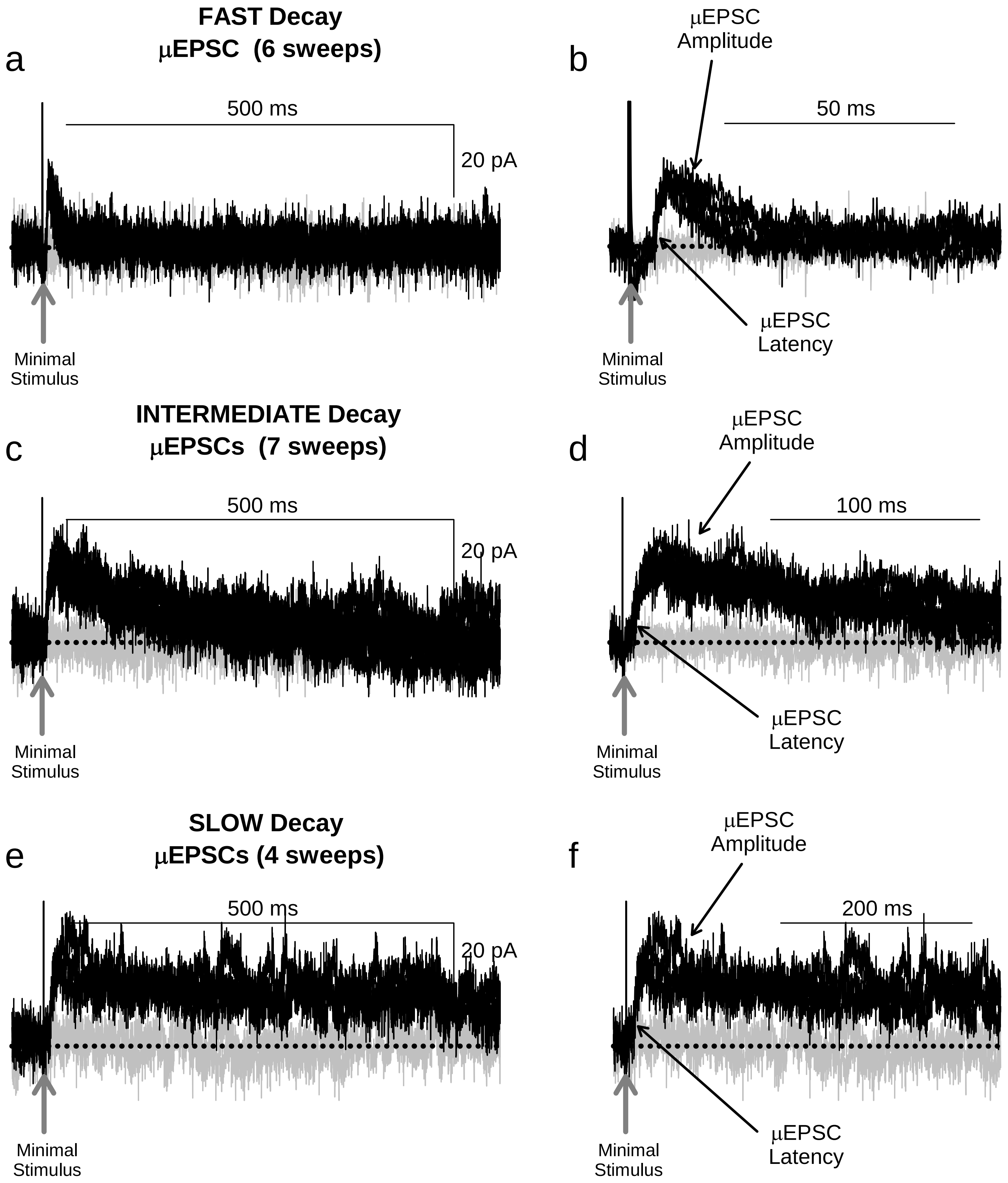

Pitcher et al., Supplementary Figure 5

a Effect of D-APV on <u>FAST</u> NMDAR μEPSC Decay

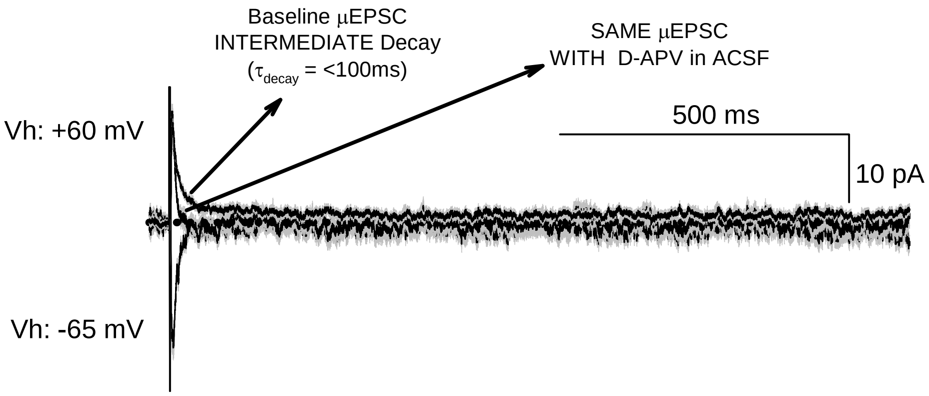

b Effect of D-APV on <u>INTERMEDIATE</u> NMDAR μEPSC Decay

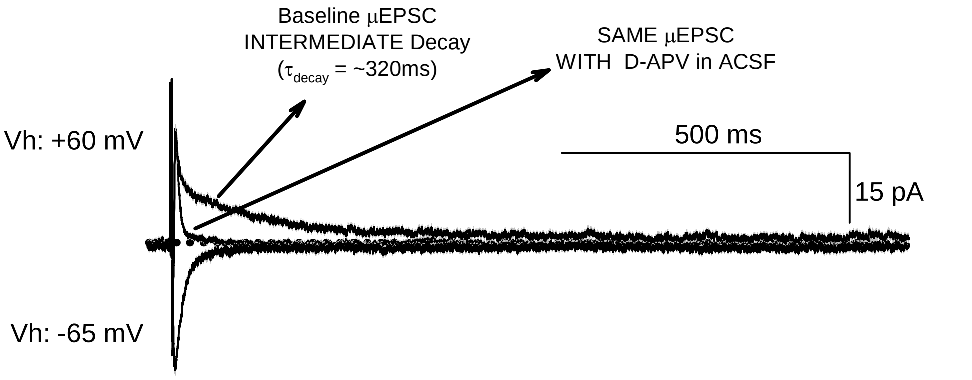

c Effect of D-APV on <u>SLOW</u> NMDAR μEPSC Decay

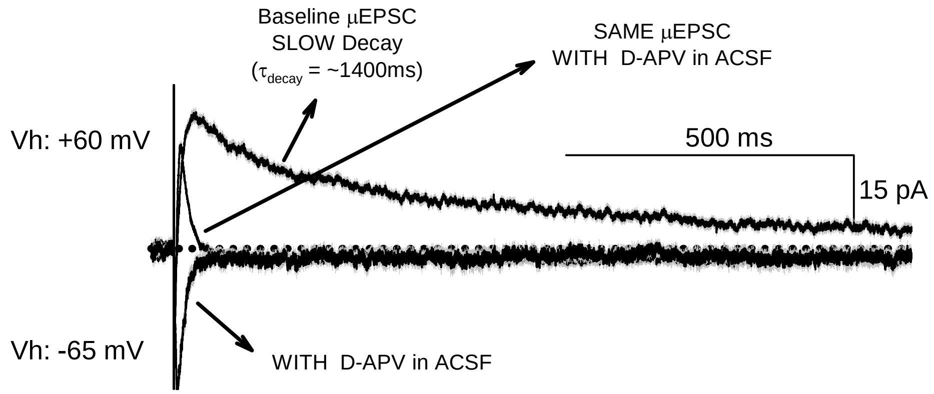

Pitcher et al., Supplementary Figure 6

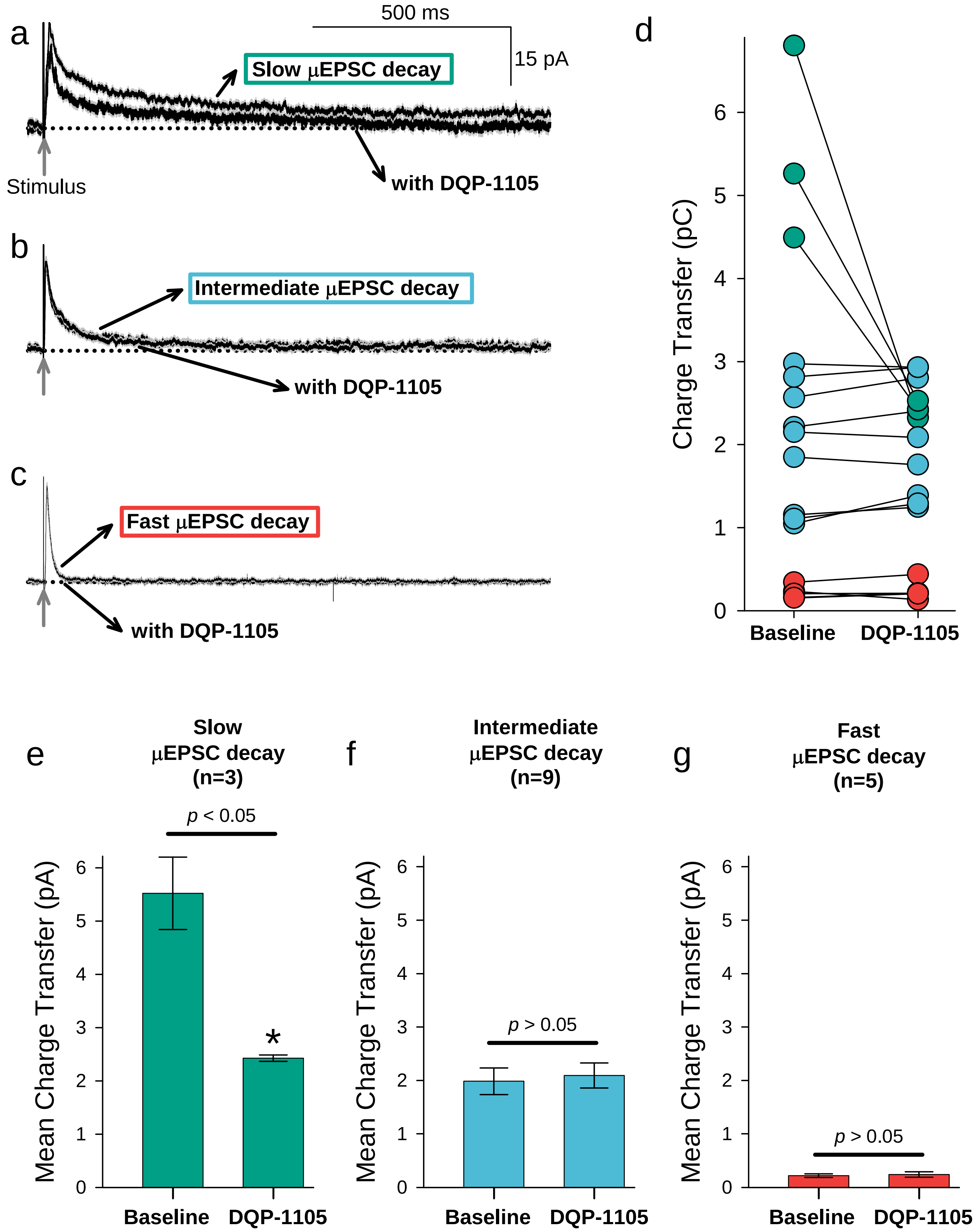

Pitcher et al., Supplementary Figure 7

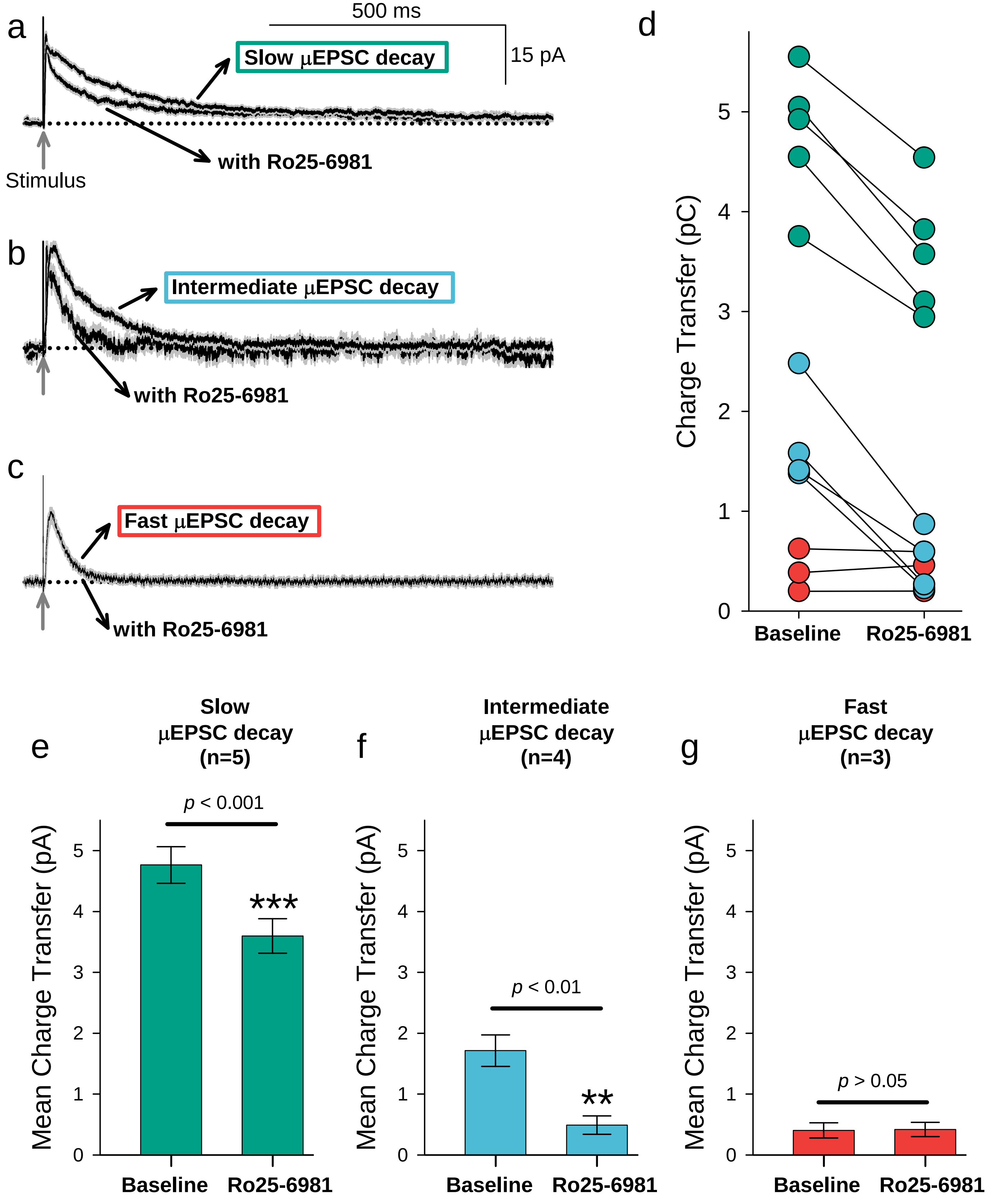

Pitcher et al., Supplemenatry Figure 8

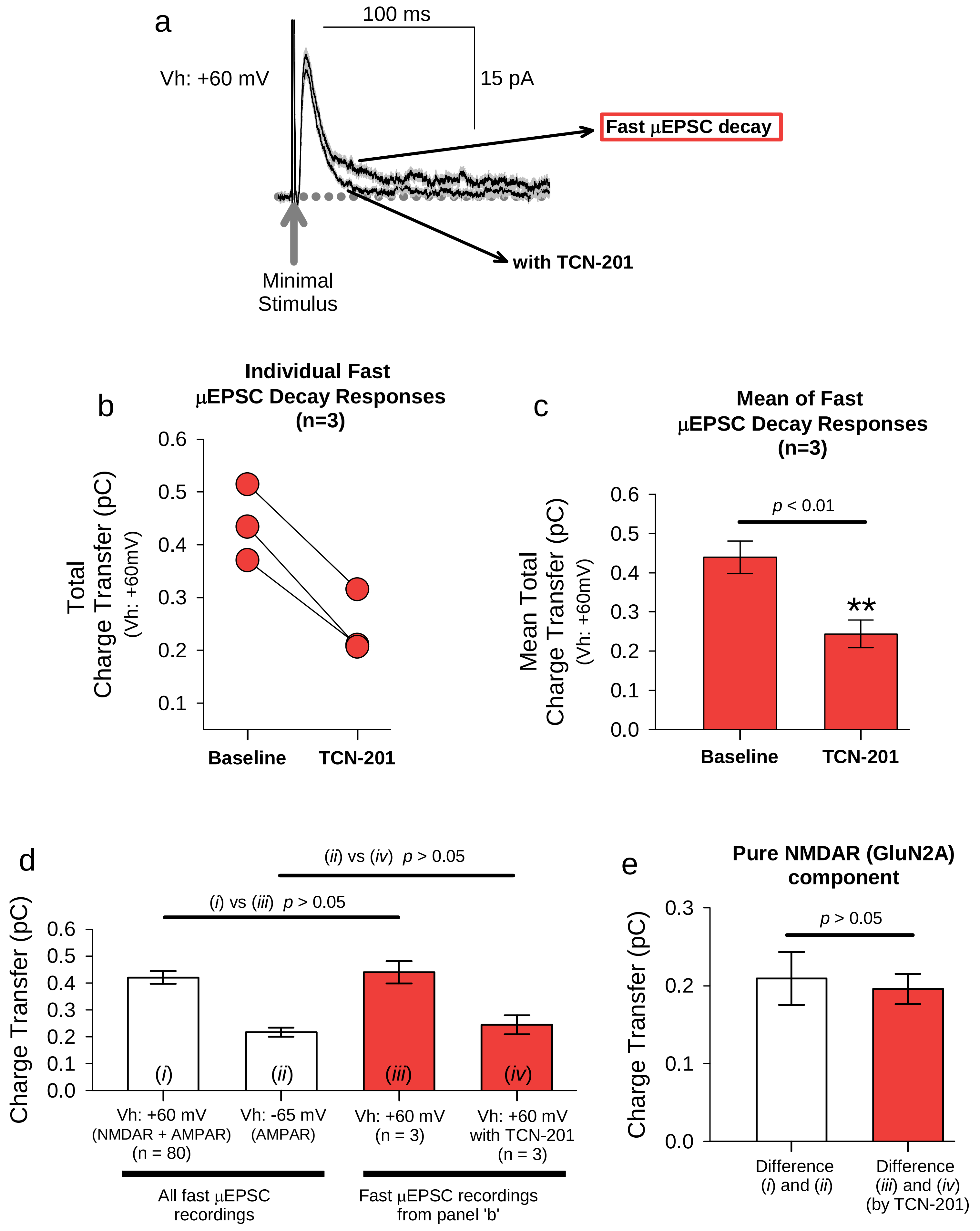

Pitcher et al., Supplementary Figure 9

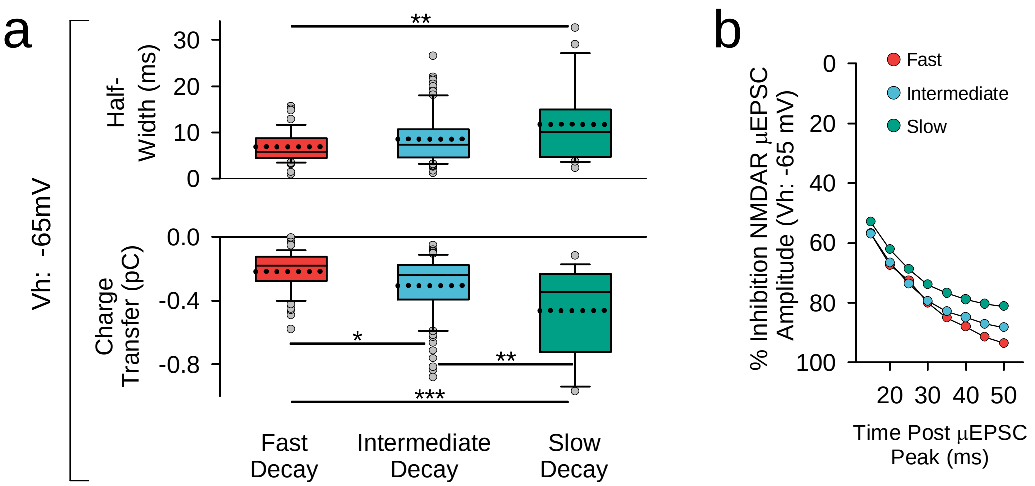

Pitcher et al., Supplementary Figure 10

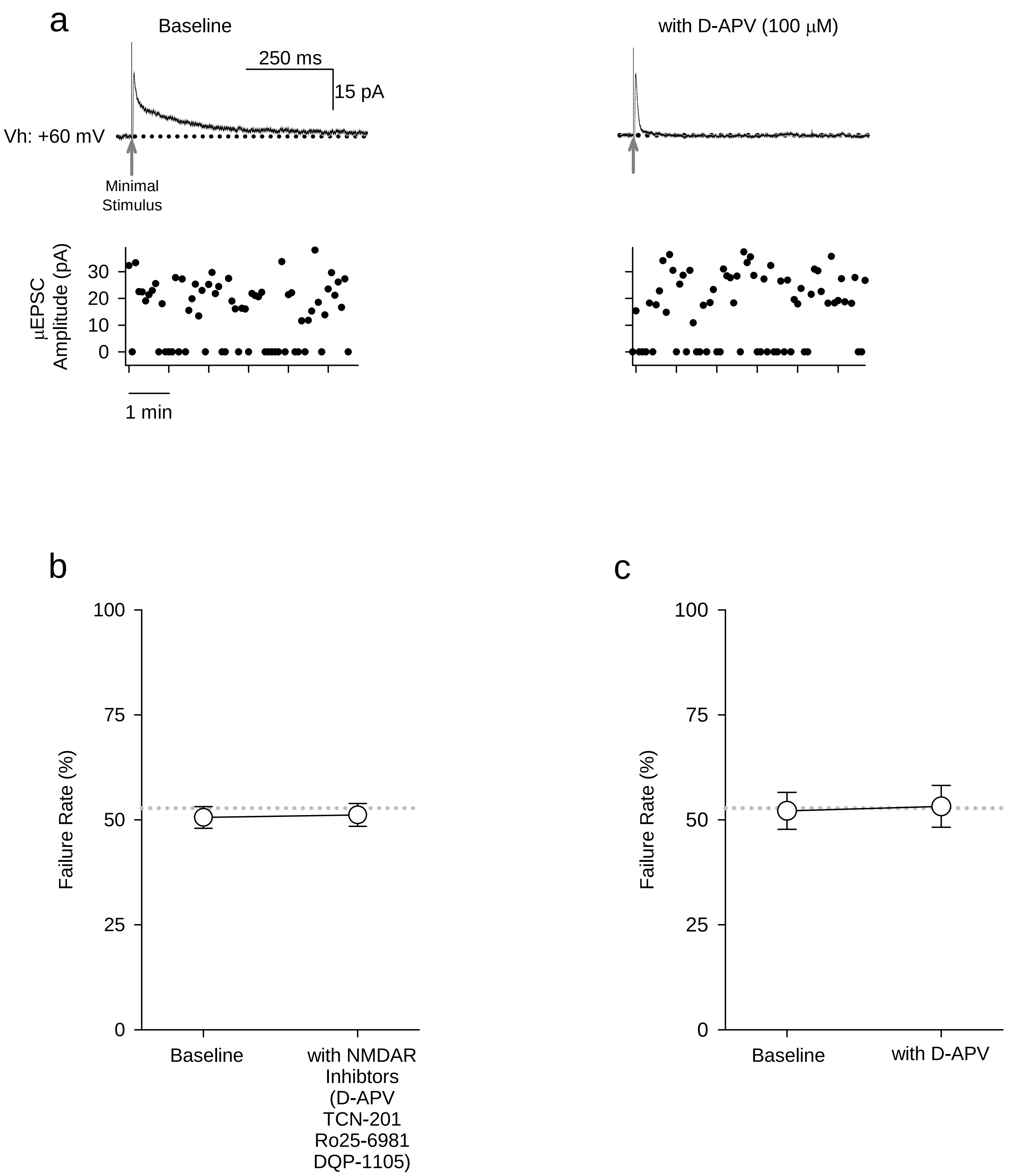

Pitcher et al., Supplementary Figure 11

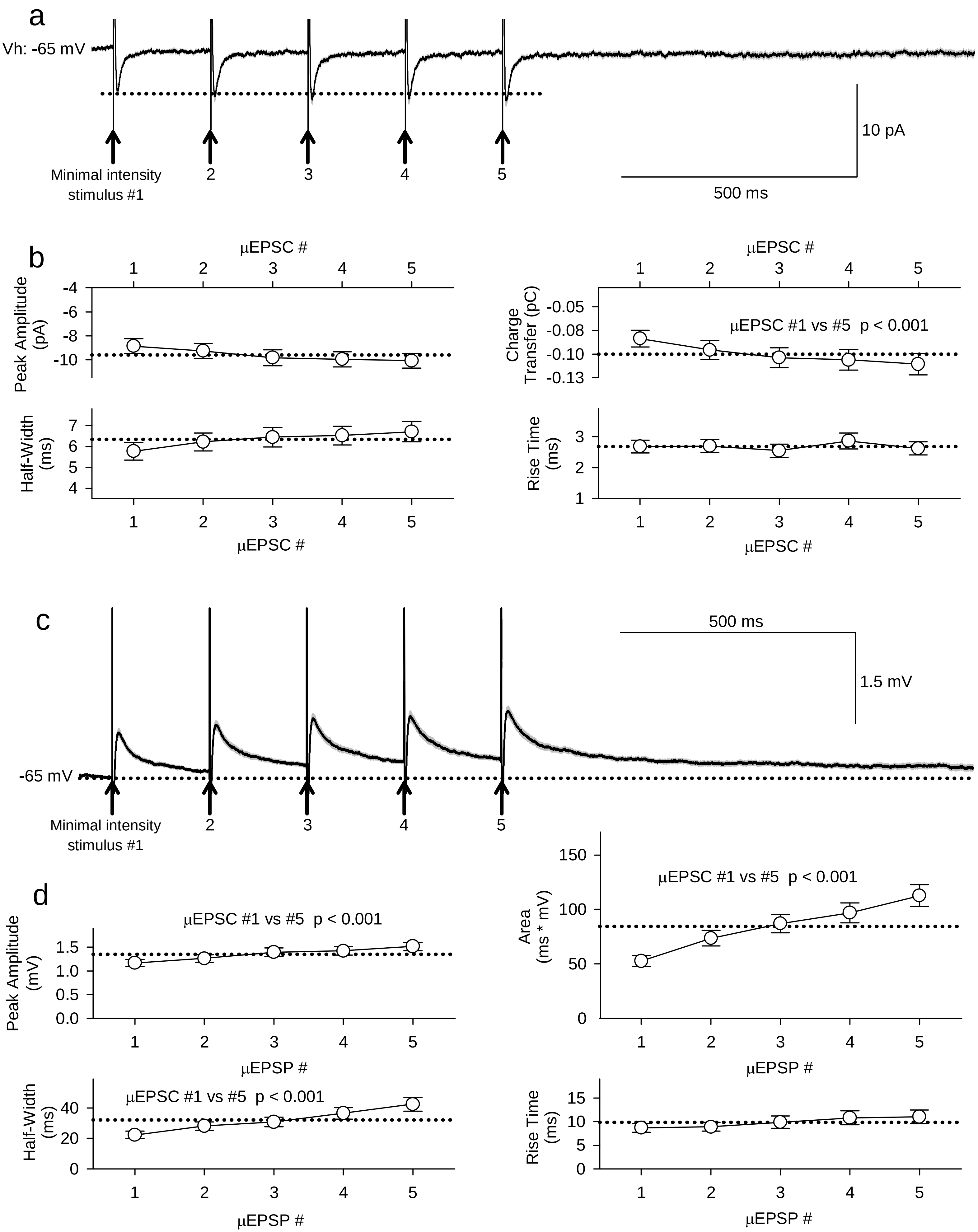

Pitcher et al., Supplementary Figure 12

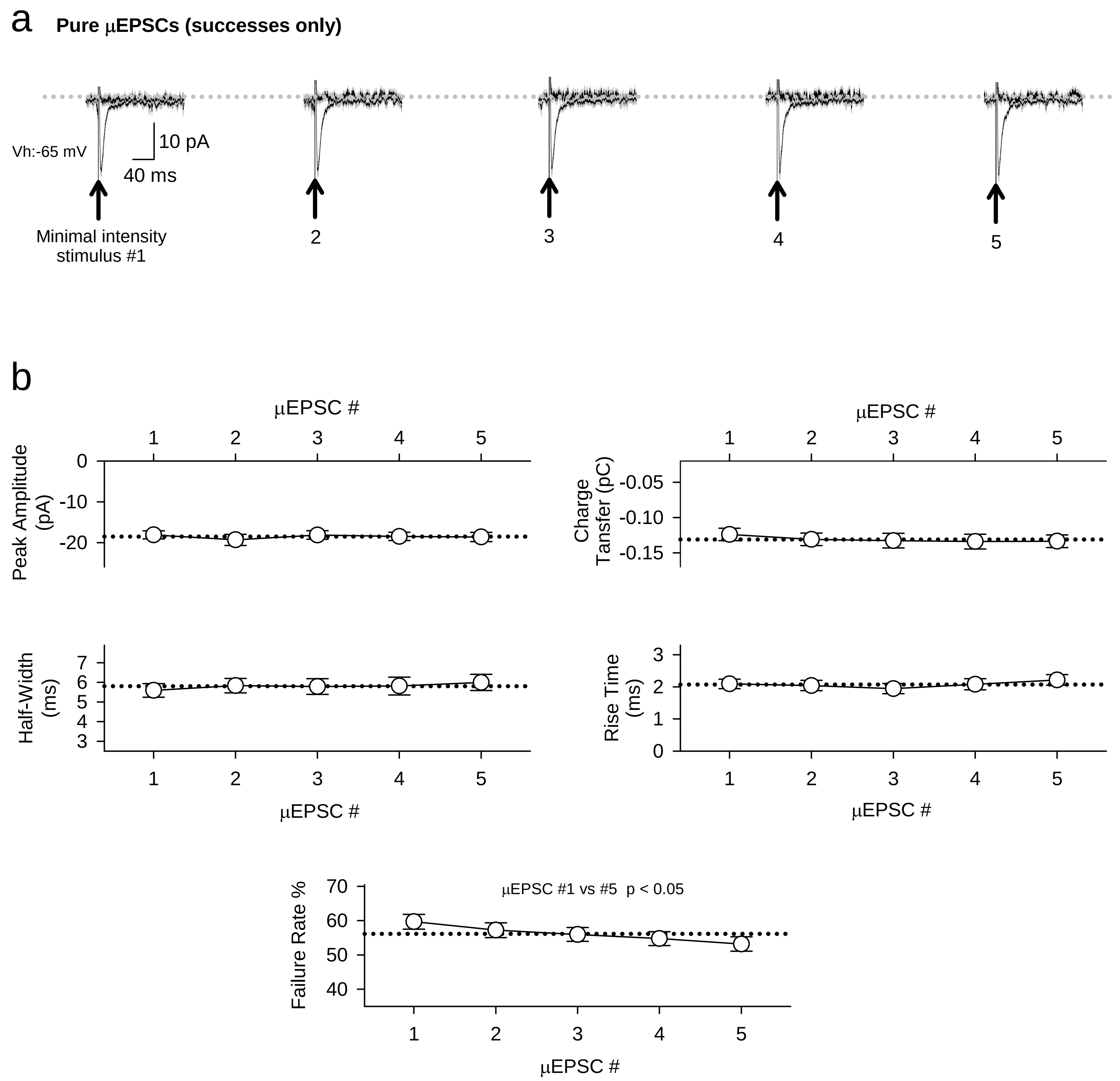

Pitcher et al., Supplementary Figure 13

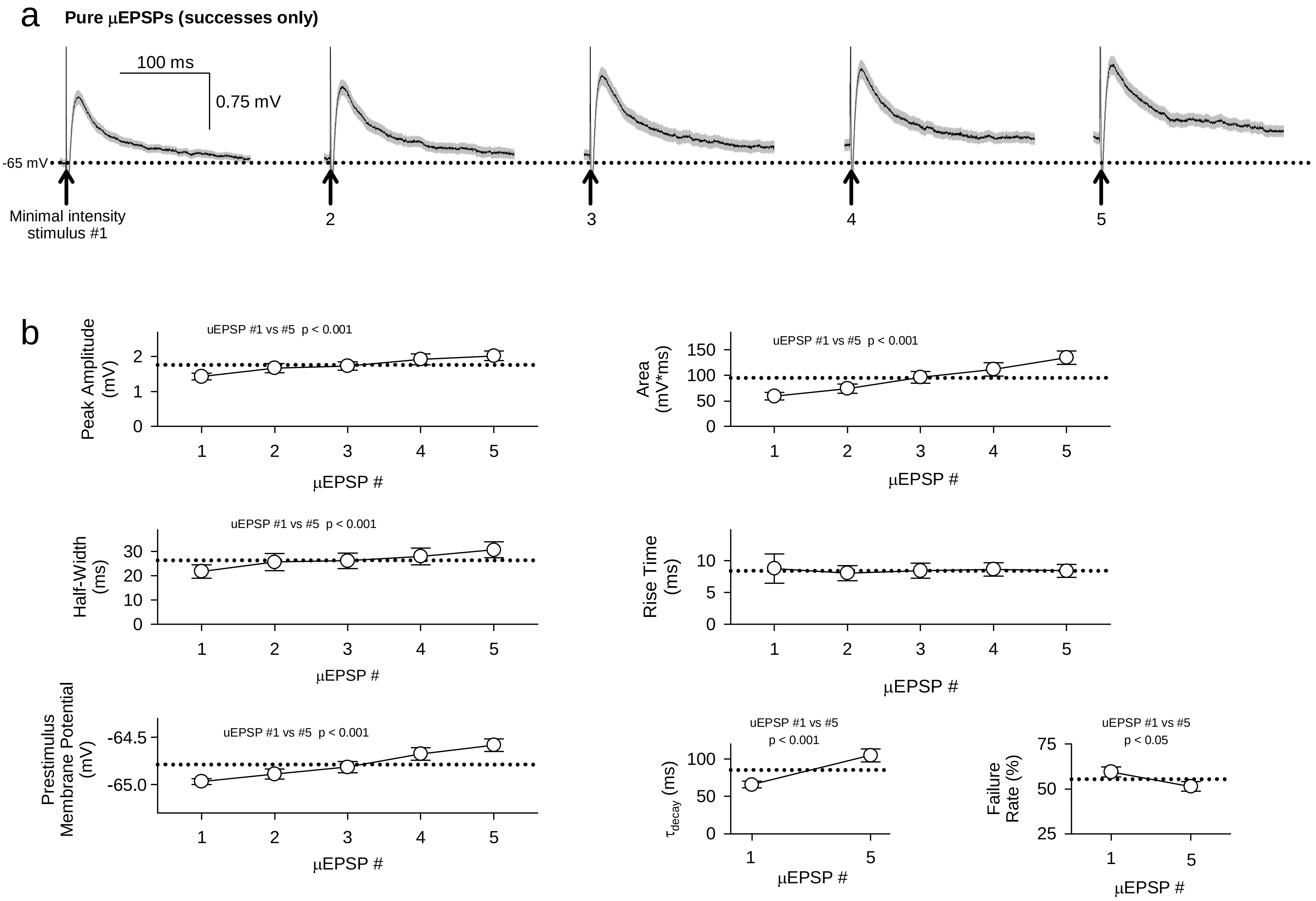

Pitcher et al., Supplementary Figure 14

a

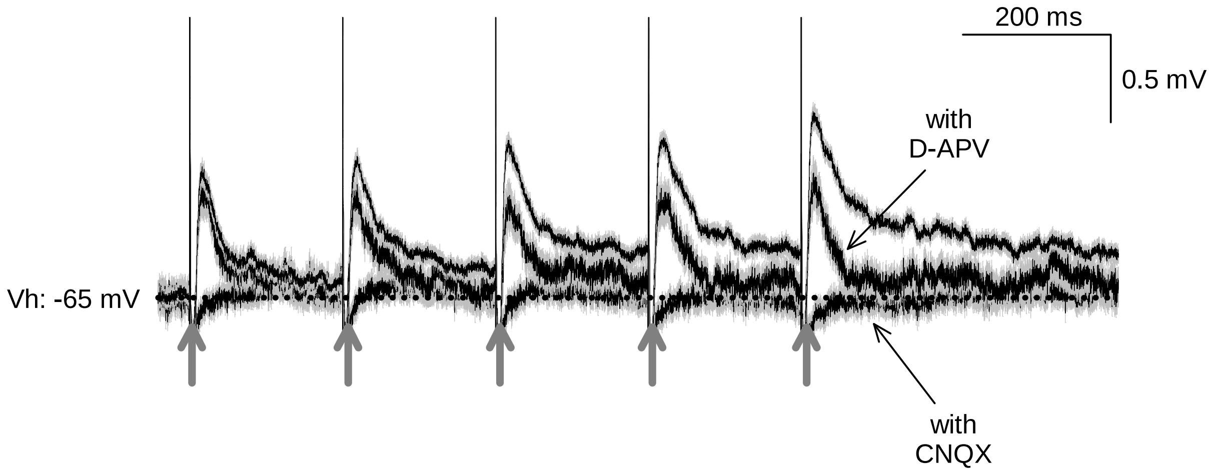

b

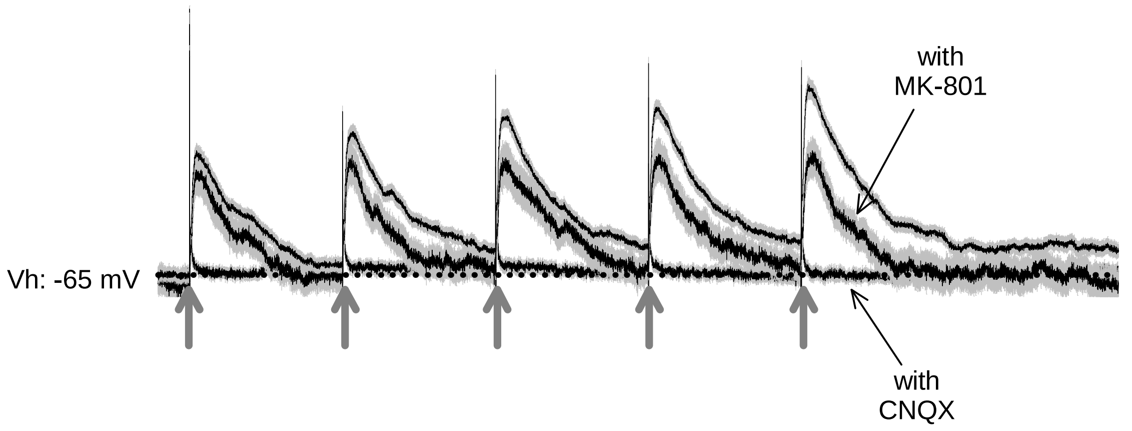

Supplement: Supplementary Figure 1 — Confirmation of minimal stimulation-evoked unitary synaptic responses. (A) Scatter plot illustrating minimal stimulation-evoked μEPSCs (Vh: −65 mv) eliminated by TTX (500 nM). (B) Left: Sample traces showing individual traces (Vh: −65 mv), (i-v) from (A), demonstrating successful μEPSCs and failures in response to minimal stimulation (the stimulus artifact is depicted by the gray arrow). A spontaneous EPSC is shown in trace iii. Right: Successful μEPSCs are blocked following TTX administration. mEPSCs are shown in traces vi-ix. (C) Scatter plot showing evoked μEPSCs (Vh: + 60 mv) at minimal stimulation threshold (gray arrow at left; sample μEPSCs shown at i-ii), and slightly above threshold (thick gray arrow at right) which evoked a second level of all-or-none responses (sample μEPSCs shown at iii-iv). Stimulus intensity below that of minimal stimulation intensity (thin gray arrow at time point 3 min) did not evoke μEPSCs. (D) Paired-pulse minimal stimulation experiment showing average amplitude (± SEM) of μEPSCs (Vh: + 60 mv) to the second stimulus [d(ii)] was greater than that of the first [d(i)] when the first stimulus also evoked a μEPSC. The average amplitude of the μEPSCs to the second stimulus [d(iii)] that followed a failure of response to the first stimulus was also identical, if not greater than, the average amplitude (± SEM) of the response to the second stimulus that followed the first stimulus non-failures. Right: d(i) scatter plot showing μEPSC amplitude with a single distribution (insert below) evoked during the first stimulus. d(ii) and d(iii) show single distributions of μEPSCs to the second stimulus. (E) Same experiment as in (d) but recorded from a different lamina I neuron. Insert above shows that single pulse minimal stimulation evoked a mean (± SEM) μEPSC recorded prior to paired-pulse stimulation (left) is identical to the mean (± SEM) μEPSC recorded in the same experiment but after paired-pulse stimulation (right). [file Data_Sheet_1.pdf]
